# In situ transformations during SLM of an ultra-strong TiC reinforced Ti composite

**DOI:** 10.1038/s41598-020-67434-3

**Published:** 2020-06-29

**Authors:** Sasan Dadbakhsh, Raya Mertens, Kim Vanmeensel, Gang Ji, Jean-Pierre Kruth

**Affiliations:** 1 PMA, Department of Mechanical Engineering, KU Leuven and Member of Flanders Make, 3001 Leuven, Belgium; 2 Department of Production Engineering, KTH Royal Institute of Technology, 10044 Stockholm, Sweden; 3 Department of Materials Engineering, KU Leuven, 3001 Leuven, Belgium; 4 CNRS, INRAE, Centrale Lille, UMR 8207 - UMET - Unité Matériaux et Transformations, Univ. Lille, 59000 Lille, France

**Keywords:** Biomedical engineering, Synthesis and processing

## Abstract

This work demonstrates a successful in situ method capable of producing an ultra-strong novel Ti composite without aluminium and vanadium. In this method, selective laser melting is used to conduct in situ alloying and reinforcing of a Ti/10.5 wt% Mo_2_C powder mixture. It is shown that this leads to a metastable β-Ti matrix homogeneously reinforced by high aspect ratio, 50–200 nm wide and up to several micrometre long TiC whiskers. The transformations of the phases are controlled by decomposition, dissolution, diffusion, and reformation of constituents. The whisker morphology of in situ formed TiC particles is associated with directional crystal growth along the TiC_<110>_ direction. The developed TiC reinforced β-Ti alloy combines a hardness over 500 HV, a Young’s modulus of 126 GPa, and an ultimate compressive strength of 1642 MPa. Improving the ductility of this composite is the subject of another work.

## Introduction

Metal matrix composites (MMCs) are intended to ideally combine the tough, ductile and conductive properties of a metal matrix with rigidity, stiffness and wear resistance of a ceramic (or in many cases intermetallics). However, this ideal combination cannot be obtained for a vast majority of the cases. This is due to several difficulties such as a weak interfacial reinforcement-matrix bonding, imperfect morphology of reinforcements, cracks at the interfaces, inhomogeneous reinforcement distributions, residual stresses, dislocations and work hardening induced by thermal mismatches between the composing phases^1^. To mitigate such issues, more research is now being directed to investigate the production of MMCs based on in situ approaches which involve chemical reactions to form reinforcements within the metal matrix. Selective laser melting (SLM) is a particularly suitable method to generate these types of MMCs. SLM is a powder based additive manufacturing (AM) method in which a complex three-dimensional part is built through laser scanning (and hence melting and solidifying) of the required geometry, layer-by-layer on top of each other. The free-form fabrication offered by SLM is an excellent opportunity for manufacturing in situ based MMCs. This enables complex geometry MMC parts with well-designed, very fine and homogenously dispersed reinforcements^2,3^, which can mitigate the common issues for this category of materials.

In contrast to previous work on in situ SLM manufacturing of MMCs from aluminium^2–6^ or steels^7,8^, here the focus is on Ti-based MMCs. Ti is one of the main metals that has been attempted to be reinforced using in situ SLM techniques. For example, the in situ reaction technique has been applied to produce bulk ceramic TiC reinforced Ti_5_Si_3_ components from a mixture of Ti and SiC with an equivalent molar ratio (according to 8Ti + 3SiC → Ti_5_Si_3_ + 3TiC)^9^. In another work, Gu et al.^10,11^ explored the reaction of 9Ti + Si_3_N_4_ → 4TiN + Ti_5_Si_3_ by SLM to obtain a composite with TiN secondary phases in an intermetallic Ti_5_Si_3_ matrix. Under optimised SLM conditions, a relative density of 97.7% is reached and the hardness drastically increased to 1358HV.

In addition to the above-mentioned powder mixtures leading to an intermetallic matrix and ceramic reinforcements by in situ reaction, there are several studies^12–14^ on SLM of ball-milled Ti-TiB_2_ powder mixtures. These investigations reported a phase change from TiB_2_ to TiB (through an in situ chemical reaction between Ti and TiB_2_^13^, i.e., Ti + TiB_2_ → 2TiB^15^). The in situ formed TiB showed a very fine needle-shape morphology and increased the hardness (~ 402 HV) and compressive strength (~ 1,103 MPa) through grain refinement and hard reinforcements in comparison with pure titanium^12–14^, but lowered the ductility.

Besides in situ reinforcing of Ti, several authors looked into in situ alloying of titanium with Mo. For example, Collins et al.^16^ were the first to mix Ti and Mo powder via laser cladding for creating compositionally graded structures. This resulted in β-Ti grains containing α-Ti precipitates. Almeida et al.^17^ also used laser cladding to synthesise Ti and 4–19 wt% Mo. After reaching a fully β-Ti alloy at about 10 wt% Mo, they reported a low Young’s modulus of 75 GPa and an adequate hardness of 240 HV at about 13 wt% Mo. More recently, Vrancken et al.^18^ looked into SLM of Ti6Al4V/10 wt% Mo powders. They found that in contrast to the fully α′ microstructure of Ti6Al4V after SLM, the Ti6Al4V/Mo microstructure consists of a β titanium matrix with residual Mo particles. The new microstructural characteristic led to a combination of a high tensile strength (*σ*_0.2_ = 858 MPa) and an excellent tensile elongation (*ε*_*frac*_ = 20.1%).

Looking at the biomedical applications of titanium-based materials, it is worth mentioning that Ti6Al4V is the most common titanium alloy to manufacture orthopaedic and dental implants (because of its combination of high strength, toughness, and corrosion resistance)^19^. Despite this popularity, there are still some long-term concerns about the potential release of harmful ions of Al and V^20,21^. In comparison, although pure Ti imposes no bio-concern over the long term biocompatibility, its lower strength and hardness makes it unsuitable for highly stressed bone implants or wear-prone prostheses^22^. Therefore, to replace Ti6Al4V and Cp-Ti for orthopaedic and dental implant applications, it is ideal to develop a hard and strong titanium alloy composite with a high hardness and strength but without any biomedical concern. To this end, future applications are looking to take advantage of solid solution strengthening elements without any toxicity concern (such as Mo^23^) or secondary strengthening phases preferably with even some bioactivity influences (such as TiC^24,25^). This can be done by using both in situ alloying and reinforcing phenomena with the assistance of the SLM process. Accordingly, this work investigates a system consists of pure Ti and molybdenum carbide (Mo_2_C). In this system, Mo_2_C is supposed to be decomposed during the SLM process and react with Ti. This occurs due to higher thermodynamic stability of the products, changing the microstructural features. This can be via formation of in situ titanium carbides while Mo dissolves into Ti as an alloying element (see Eq. 1). For a better understanding, this system is compared to pure Ti. According to the microstructural characteristics and the mechanical properties of the manufactured components, the formation and growth mechanisms of in situ products are investigated and discussed.

1 $${\text{Ti}} + {\text{Mo}}_{{2}} {\text{C}} \to {\text{TiC}} + {\text{Ti}}\left( {{\text{2Mo}}} \right)$$

## Results

### Cross-sectional analysis

**Figure 1:**
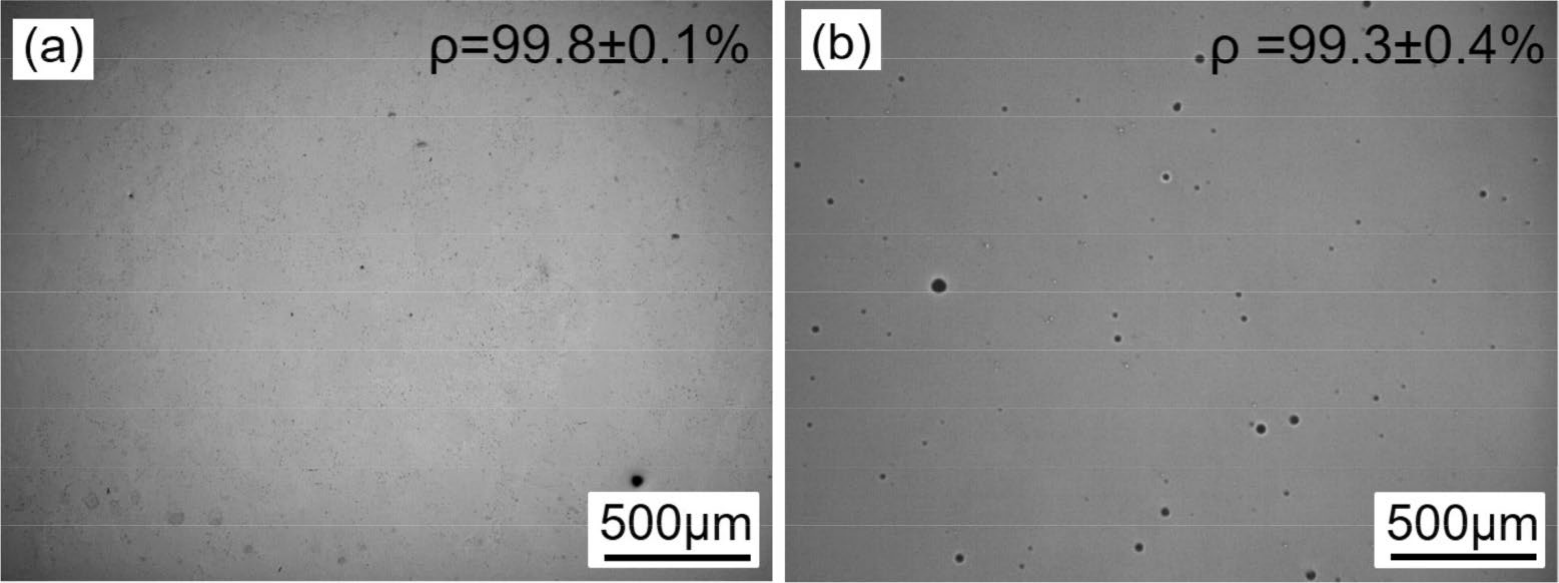
Relatively dense cross-sections of optimally dense and crack-free parts from (**a**) Ti and (**b**) Ti/ Mo_2_C. The samples are cut along building direction (BD).

Pure Ti theoretical density is about 4.51 g/cm^3^^26^. This was much less than the density of the current material, as measured to be 4.75 ± 0.05 g/cm^3^ from the Archimedes principle. This was since pure Ti is α-phase at room temperature without any additional combination, while the in situ material developed here was no longer an α-Ti and contained Mo and in situ secondary particles. Therefore, since no straightforward theoretical density was available for this new material, no accurate relative density could be calculated by measuring the density. Therefore, analysing the cross section were found to be the best method to estimate density, as shown in Fig. 1. This demonstrates that crack free parts were produced from both material systems (Ti and Ti/Mo_2_C) using optimal SLM parameters, where the remaining porosity (< 1%) appeared to be spherical in both cases. However, Ti SLM parts were denser (~ 99.8% vs. 99.3%), which is logical considering the simpler system and the better powder flow of pure Ti. Moreover, the visually larger pore sizes in the composite system in the range of 5–70 µm (typically ~ 30–40 µm) may impose a stronger effect to reduce ductility compared to small pores below 5 µm in Cp–Ti. Nevertheless, from the cross sectional analysis, the theoretical density of the current material seem to be around 4.80 ± 0.03 g/cm^3^.

### XRD

**Figure 2:**
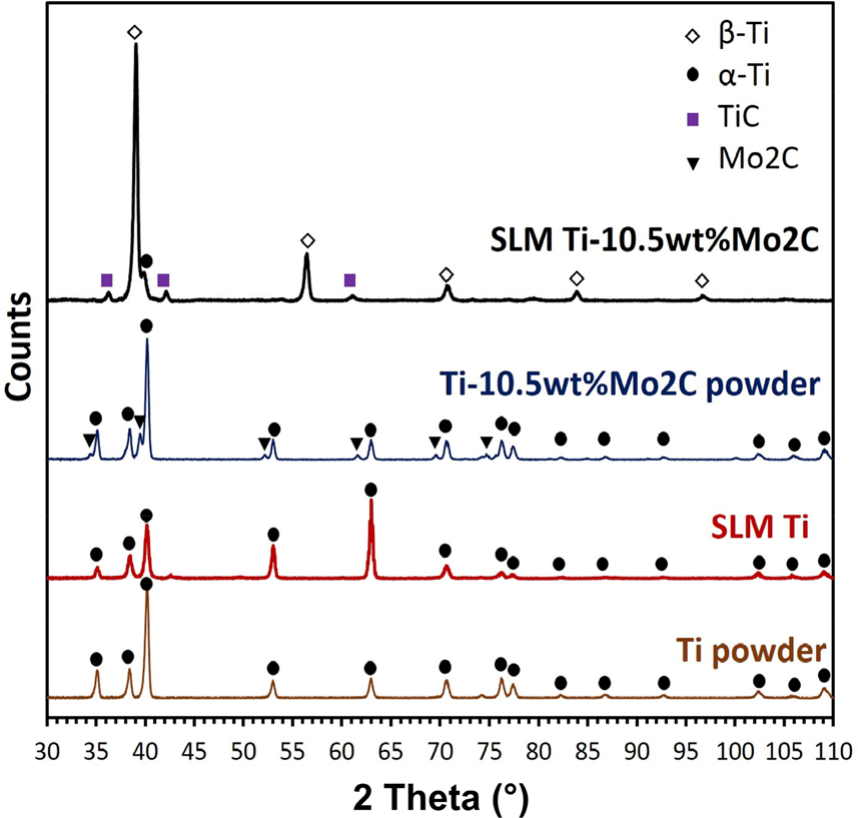
XRD spectra of Ti powder, Ti/10.5 wt% Mo_2_C powder mixture, and the corresponding SLM parts.

The XRD patterns of the Ti and Ti/10.5 wt% Mo_2_C powders and the corresponding SLM parts are shown in Fig. 2. As seen, the α-Ti peaks representative for the hexagonal Ti powder are maintained after SLM. In contrast, when comparing the diffraction spectra of the Ti/10.5 wt% Mo_2_C powder and part after SLM, it can be observed that the intensity of the hexagonal α-Ti peaks was significantly reduced in favour of the formation of a β-Ti phase with cubic crystal structure. Additionally, no Mo_2_C peaks were detected and instead a small fraction of TiC was observed after SLM. This can indicate that the initial Mo_2_C had completely decomposed in the melt pool during SLM. After Mo_2_C decomposition, the resulting Mo incorporated into the Ti lattice, stabilising the BCC β-Ti crystal structure, while carbon was preferentially reacting with Ti, forming TiC. A small fraction of α-Ti phase is still detected after SLM since the Mo content in the powder mixture (9.88 wt%) is slightly below the critical content required to fully stabilise the β-Ti phase (10 wt%^17^).

### Microstructural analysis

#### Microstructural appearance after SLM

**Figure 3:**
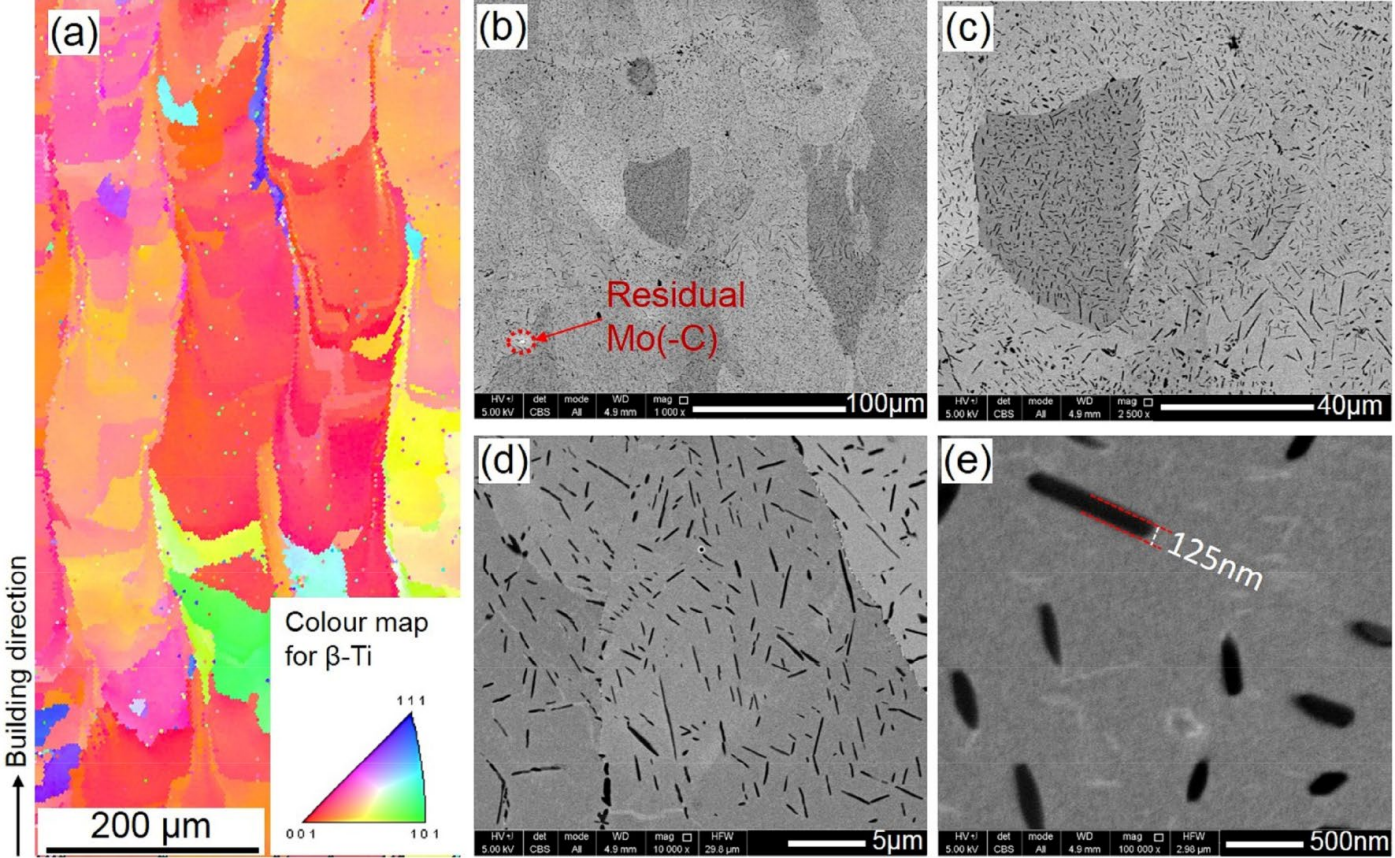
High resolution SEM and EBSD pictures of Ti/Mo_2_C SLM parts using optimal parameters. (**a**) EBSD image of the grain structure, largely elongated in the building direction (vertical and upwards), (**b**) dominant granular microstructure containing (**c**–**e**) homogenous distribution of nano whisker-shape carbides (as observed with the black contrast).

It is well reported that pure Ti after SLM has an acicular and lath-like α΄ (martensitic α-Ti) microstructure^27,28^. The addition of Mo_2_C, however, completely changes the microstructural characteristics, as shown in Fig. 3. As Mo stabilises the high temperature β-Ti phase, the EBSD map, shown in Fig. 3a, indicates that the material mainly consist of the cubic β-Ti phase after SLM. Additionally, as the EBSD map was collected within a plane parallel to the building direction, a predominant <100> texture is observed, corresponding to the preferential growth direction of BCC metals during solidification. No martensitic α′ phase with hexagonal crystal symmetry and acicular shape was detected in contrast to SLM processed pure Ti^27,28^. Figure 3b–e indicate that a secondary phase is homogeneously dispersed within the Ti matrix after SLM. The secondary phase particles content add up to 6 vol%, based on image analysis. Morphologically, they have a whisker shape with a width of typically less than 200 nm and a length that can reach several micrometres (Fig. 3c–e). Occasionally, a small amount of unreacted Mo_2_C–Mo may remain after SLM, as observed in Fig. 3b.

#### In situ reaction footmarks

**Figure 4:**
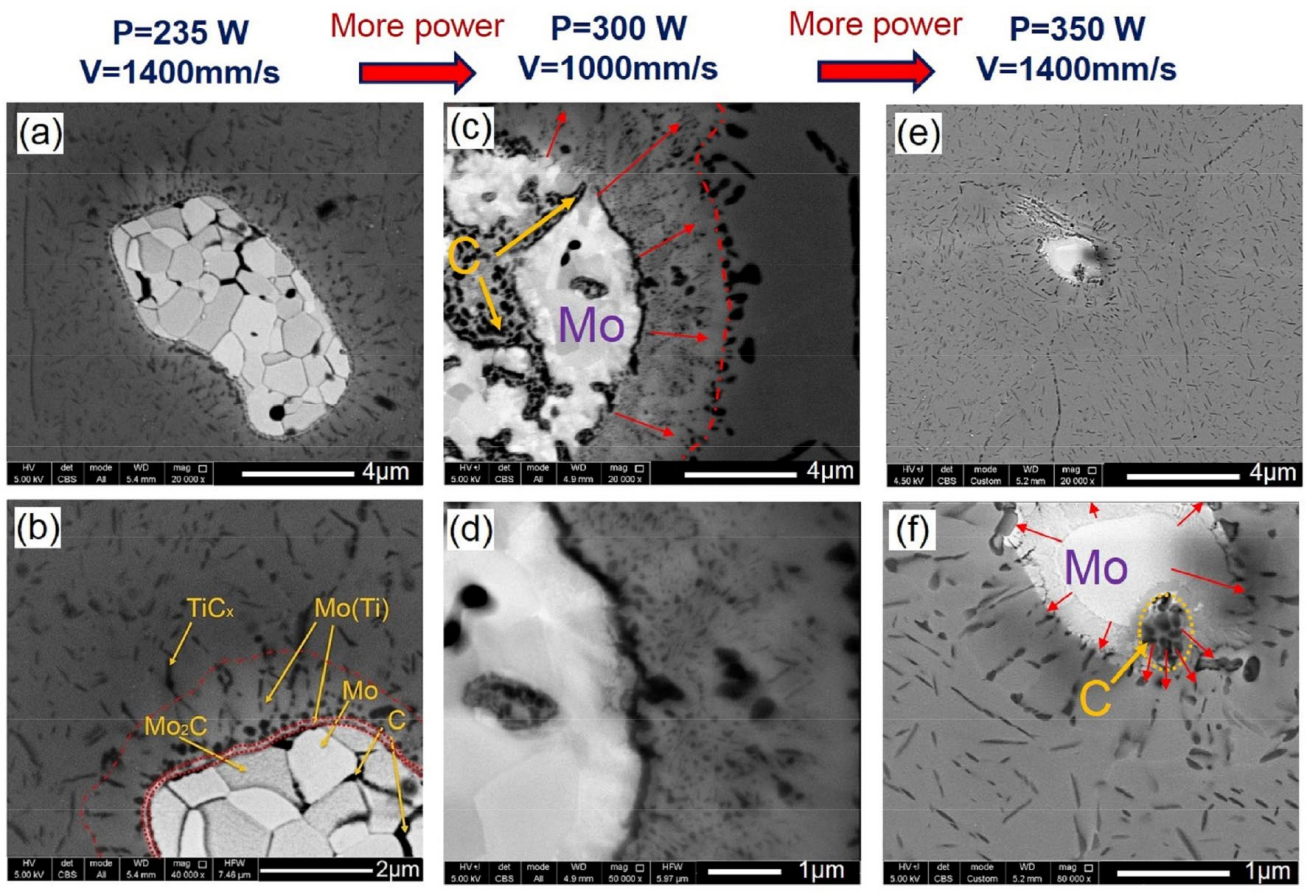
SEM pictures (with BSE contrast) of residual Mo/Mo_2_C particles in SLM parts made from Ti/Mo_2_C using different laser parameters, illustrating the in situ transformations. (**a**, **b**) The initial interaction of a Mo_2_C particle with the molten Ti matrix during SLM at a low laser energy, where a Mo_2_C polycrystalline particle is partitioning to the original crystals and decomposing to Mo and C. (**c**, **d**) Accumulation of nano-sized C inside Mo while Mo and C are gradually migrating into the Ti matrix. (**e**, **f**) a small residual Mo particle dissolving into Ti matrix and releasing the accumulated carbon particles. The red arrows indicate the direction of diffusion and migration. The orange arrows are for identifying the different phases.

In order to elucidate the in-situ reaction mechanism, the residual Mo_2_C/Mo particles (see Fig. 3b) and their immediate surroundings were investigated in detail, representing the actual freeze frames of the dissolved particles. Figure 4 shows the transformation evolution through an increase in laser power. As shown in Fig. 4a, b, taken from an as-built sample processed at low laser energy, a residual Mo_2_C particle consisting of several grains is still present within the Ti matrix. In these pictures, Mo appears with the brightest colour, attempting to penetrate into the Ti matrix. Next to Mo, Mo_2_C particles (more greyish in contrast to white Mo) may exist, as a source of carbon, releasing black carbon into the crystalline borders. At higher laser power (Fig. 4c, d), carbon accumulates and coalesces in residual Mo pieces until it finds the chance to be released while Mo is being dissolved into the Ti matrix. After releasing of carbon into Ti, they diffuse within the melt and form whisker-shaped carbides. This continues until almost all the Mo dissolves, leaving only few carbon particles to migrate into Ti matrix (as it can be seen from higher powers in Fig. 4d, f). It should be reminded that the black particles are from carbon and not pores, since they have a clear diffusion tendency and they change their form when they diffuse into the Ti matrix from Mo.

### Mechanical properties

**Figure 5:**
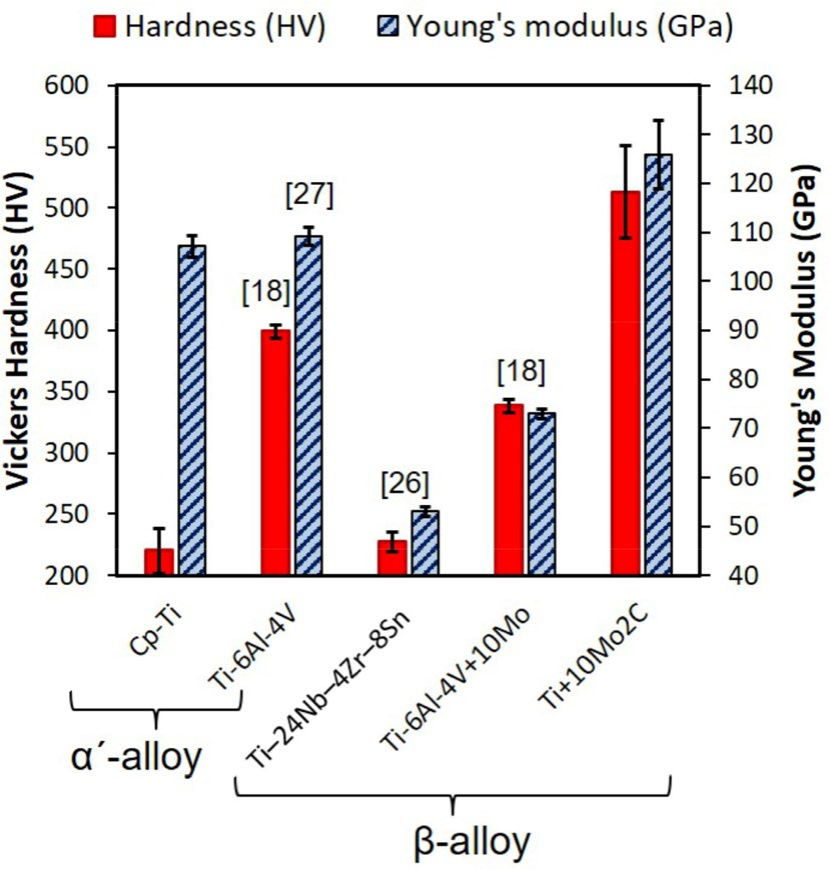
Hardness and Young’s modulus of the composite made in this work compared to other SLM processed Ti alloys.

Micro Vickers tests on SLM Ti/Mo_2_C parts showed a hardness of 513 ± 38 HV (equal to 50 ± 3 HRC). This is significantly higher than the hardness of SLM Cp-Ti (220 ± 19 HV), SLM Ti-6Al-4 V (399 ± 5 HV^18^) as well as β-alloy SLM Ti–24Nb–4Zr–8Sn (227 ± 8 HV^29^) and Ti-6Al-4 V + 10Mo (338 ± 5 HV^18^), as summarised in Fig. 5. The Young’s moduli of SLM Ti and Ti/Mo_2_C, as measured using IET at room temperature, were determined to be 107 ± 2 GPa and 126 ± 7 GPa, respectively. Therefore, SLM of Ti/Mo_2_C powder resulted in the hardest and the stiffest Ti alloy amongst the most common Ti alloys (Fig. 5).

**Figure 6:**
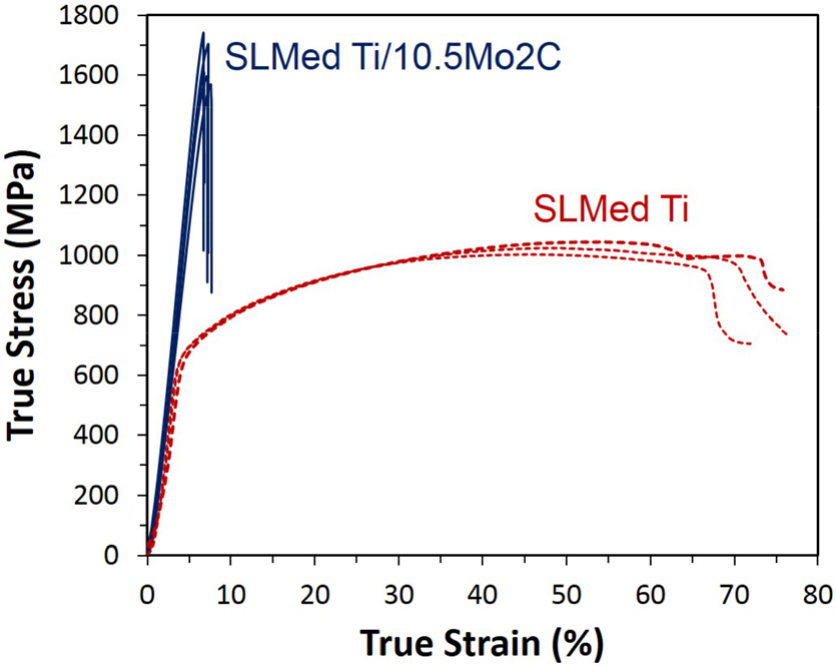
Compressive stress–strain curves of pure Ti and Ti/Mo_2_C SLM parts. Note that the compressive displacements of the compression plates are also included in the reported compressive strains.

Figure 6 shows the compression curves of the SLM parts made from Ti/Mo_2_C alloy. The SLM parts can reach a very high compression strength of 1642 ± 77 MPa, but they fail without any plastic deformation. This is very different from the curves of pure Ti showing a much lower compressive yield and ultimate strength, but much higher ductility. The recorded compressive strains included all the compressive displacements from samples and compression plates and therefore they can be used only for comparison.

**Figure 7:**
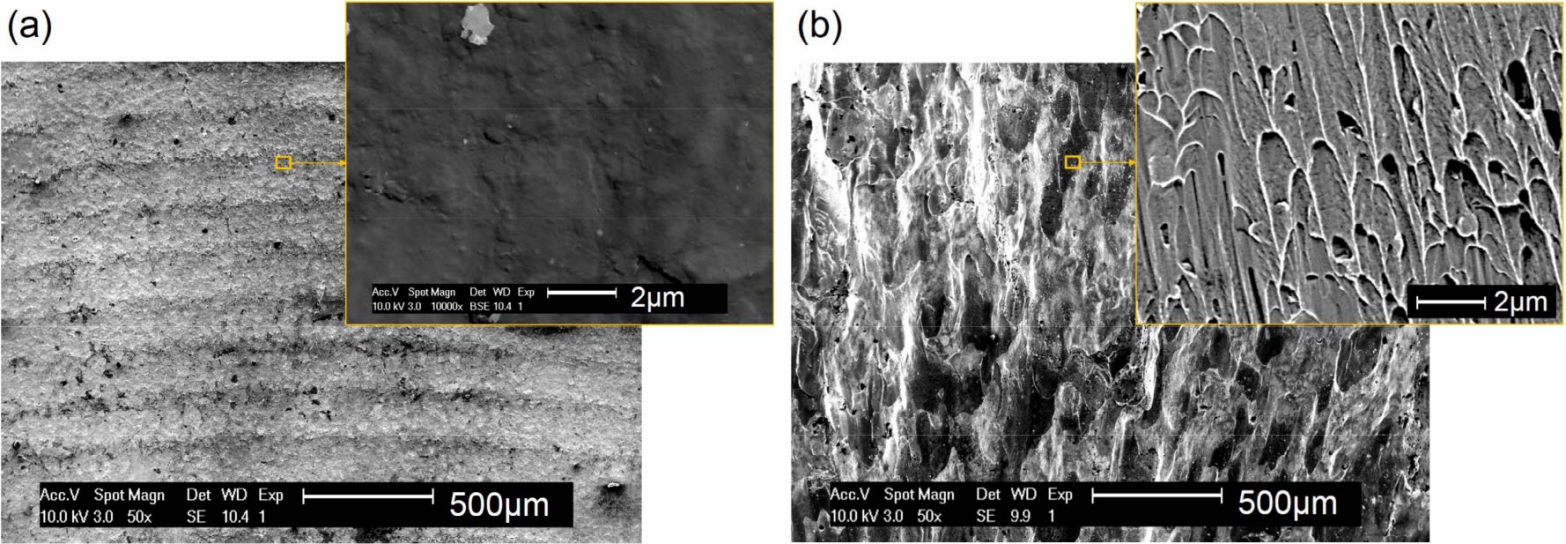
Compressive fracture surfaces of (**a**) Ti and (**b**) Ti/Mo_2_C SLM parts.

Figure 7 shows the fracture surfaces after the compression tests of pure Ti and Ti/Mo_2_C parts. As seen, compressive fracture in Ti has developed relatively smoothly, even from a close view (Fig. 7a). In contrast, the rougher fracture surface of the Ti/Mo_2_C sample hints at a more brittle behaviour. Additionally, the presence of fine cavities, which are observed on the fracture surface, especially near the ends of the solidified grains (Fig. 7b), can be due to whisker particles pull-out. It can be argued that the presence of carbide whiskers hinders material shear and hence plastic flow.

## Discussion

### In situ formation mechanisms

**Figure 8:**
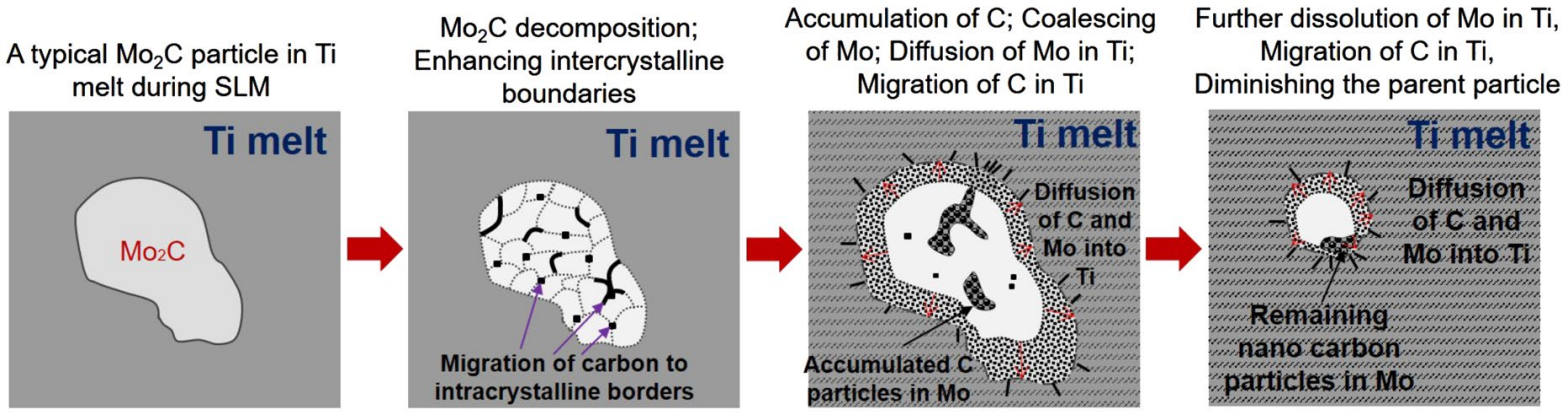
Decomposition and diffusion mechanisms diminishing the parent Mo_2_C particles and reinforcing Ti.

For successful SLM of an in situ reacting powder mixture, it is very important to understand the transformation mechanisms of the constituents. This is particularly interesting since the melting point of Mo_2_C is very high (~ 2,687 °C) which cannot normally be reached during SLM with the employed parameters. Therefore, in accordance to the changes of the residual Mo/Mo_2_C particles in Fig. 4, Fig. 8 schematically explains how the in situ transformation occurs. As seen, with raising temperature in Ti solid/melt, a typical Mo_2_C particle (Fig. 8a) decomposes due to more stability of dissolved Mo in liquid Ti and Ti carbide at high temperatures. More specifically, for the corresponding composition of 0.0265 mol Mo_2_C and 0.9735 mol liquid Ti at 2000 °C, the Gibbs free energy of Mo dissolution in liquid Ti is − 17.9 kJ (calculated using Thermo-Calc Software, TCTI2 database, 2019) and TiC formation is − 4.1 kJ against only − 1.6 kJ stability of Mo_2_C (calculated from the Gibbs free energy graphs in ^30^). The resulting Mo_2_C decomposition occurs by splitting Mo_2_C into several crystalline Mo-rich regions while accumulating the C within crystalline borders (Fig. 8b). At the same time with the migration and accumulation of carbon nano-particles at the intercrystalline Mo_2_C borders, Mo regions coalesce until the borders between them diminish. This is followed by accumulation and coalescence of carbon inside Mo regions. Now after these decomposition phenomena, logically solid Mo (T_m_ ~ 2,623 °C) and C (T_m_ ~ 3,550 °C) can both diffuse to the Ti melt (Fig. 8c). While C is being combined with Ti to form titanium carbides, Mo dissolves in Ti melt (Fig. 8d). After solidification, Mo saturated Ti lead to a β-Ti matrix (due to rapid solidification of Ti–10Mo) reinforced by numerous, fine, and homogeneously dispersed TiC particles (see Figs. 2, 3).

**Figure 9:**
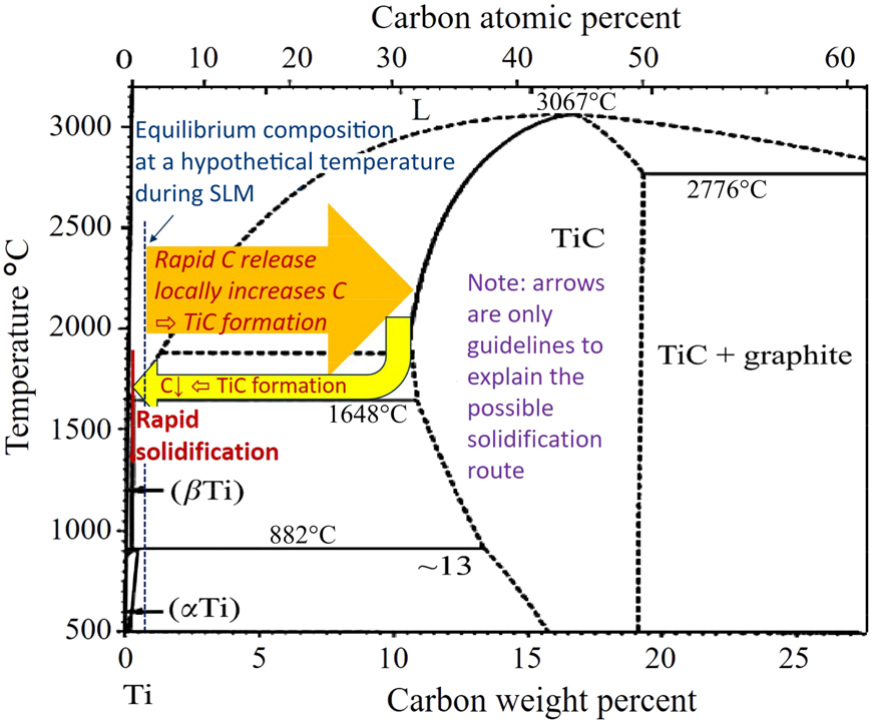
Schematic of the possibility of hypereutectic TiC formation after sudden release of carbon prior to the rapid solidification. The equilibrium Ti-C diagram is adapted with permission from ^34^.

It worth mentioning that this theory more looks into the possibility of TiC formation through a hypereutectic reaction (Fig. 9). This is also in confirmation with the previous studies on melting of Ti–15Mo–0.2C, reporting the formation of coarse TiCx from the melt due to the role of Mo to reduce the carbon dissolubility in Ti^31^. In addition to this for the current case, it seems that after a rapid release of carbon, TiC forms due to a local high carbon content around the carbon particles. This is in agreement with the kinetic studies on Ti–C systems, concluding that instead of dissolving C in Ti, TiC primarily forms below the combustion temperatures of 2,438 °C^32^. This is also the main temperature range in a Ti melt in which a successful SLM occurs^33^. Above this temperature, which could be reached perhaps using excessively high laser energies, carbon dissolution becomes easier into the titanium melt (instead of TiC formation)^32^, forming TiC from the eutectic reaction. However, these high temperatures (e.g., above 2,500 °C) could not be dominantly reached using the employed optimal parameters for SLM at this work, since excessive over melting has been avoided here.

**Figure 10:**
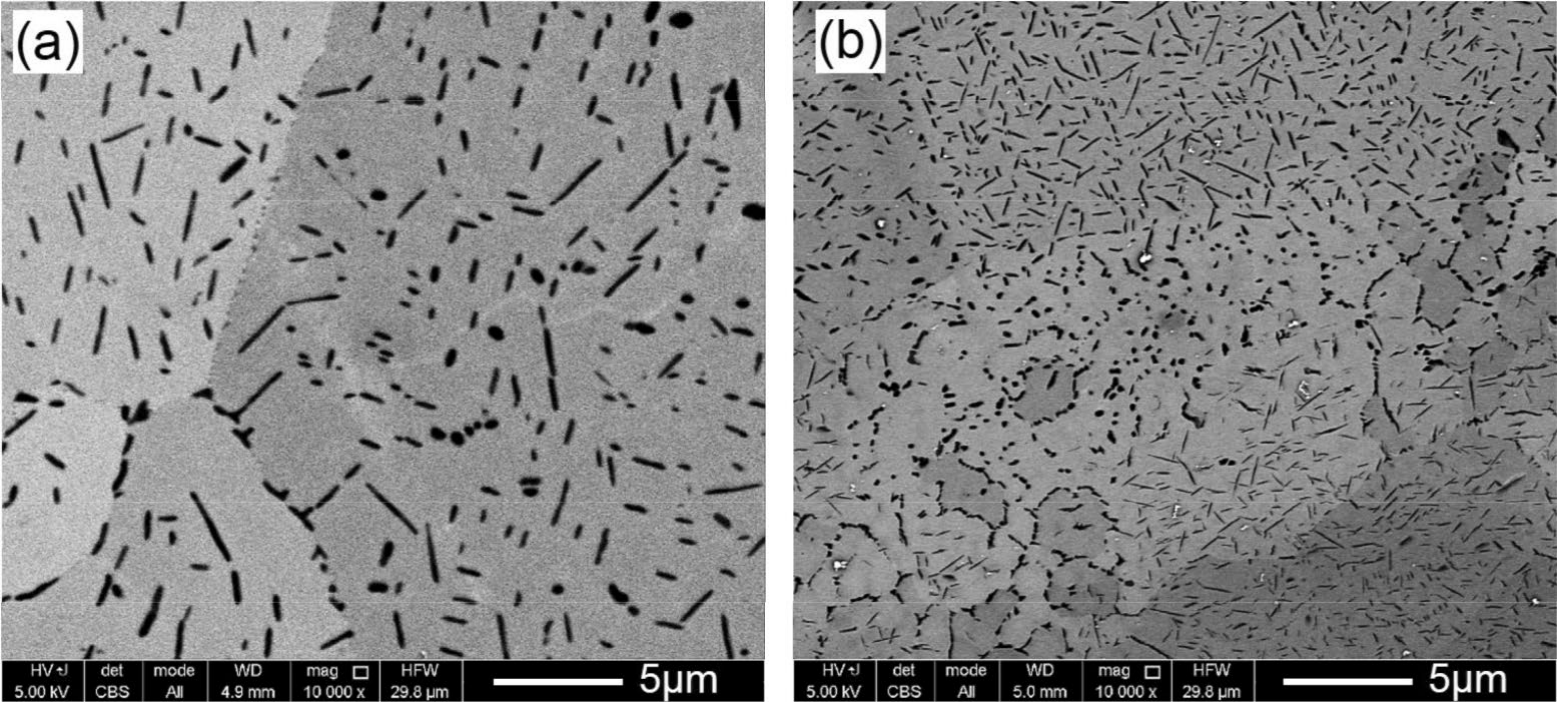
Microstructural refinement and precipitations in cellular format with decreasing the laser scanning speed from (**a**) 1,000 mm/s (and P = 300 W) to (**b**) 100 mm/s (and P = 50 W). Notice that this is contradictory to the microstructural coarsening at a lower scanning speed.

According to the above-mentioned theory, the fast solidification at high laser scanning speeds has a determining effect to suppress dissolution of TiC particles after formation. As a result, when the scanning speed is decreased, TiC particles become thinner and shorter (since the formed TiC thermodynamically tends to dissolve in the Ti melt, as seen in the binary diagram in Fig. 9, utilising the longer-lasting melt). This is while eutectic TiC increases (compare Fig. 10a and b showing the effects of the scanning speed reduction). This significantly refines and changes the microstructure, as the eutectic TiC can be finer or may even precipitate in a cellular format (Fig. 10b). It should be noted that this is contradictory to the well-known refining influence of the higher solidification rates. This is since the formation mechanisms are completely changed here (from hypereutectic to eutectic) with the use of much lower laser speeds.

### In situ growth and crystal orientations

**Figure 11:**
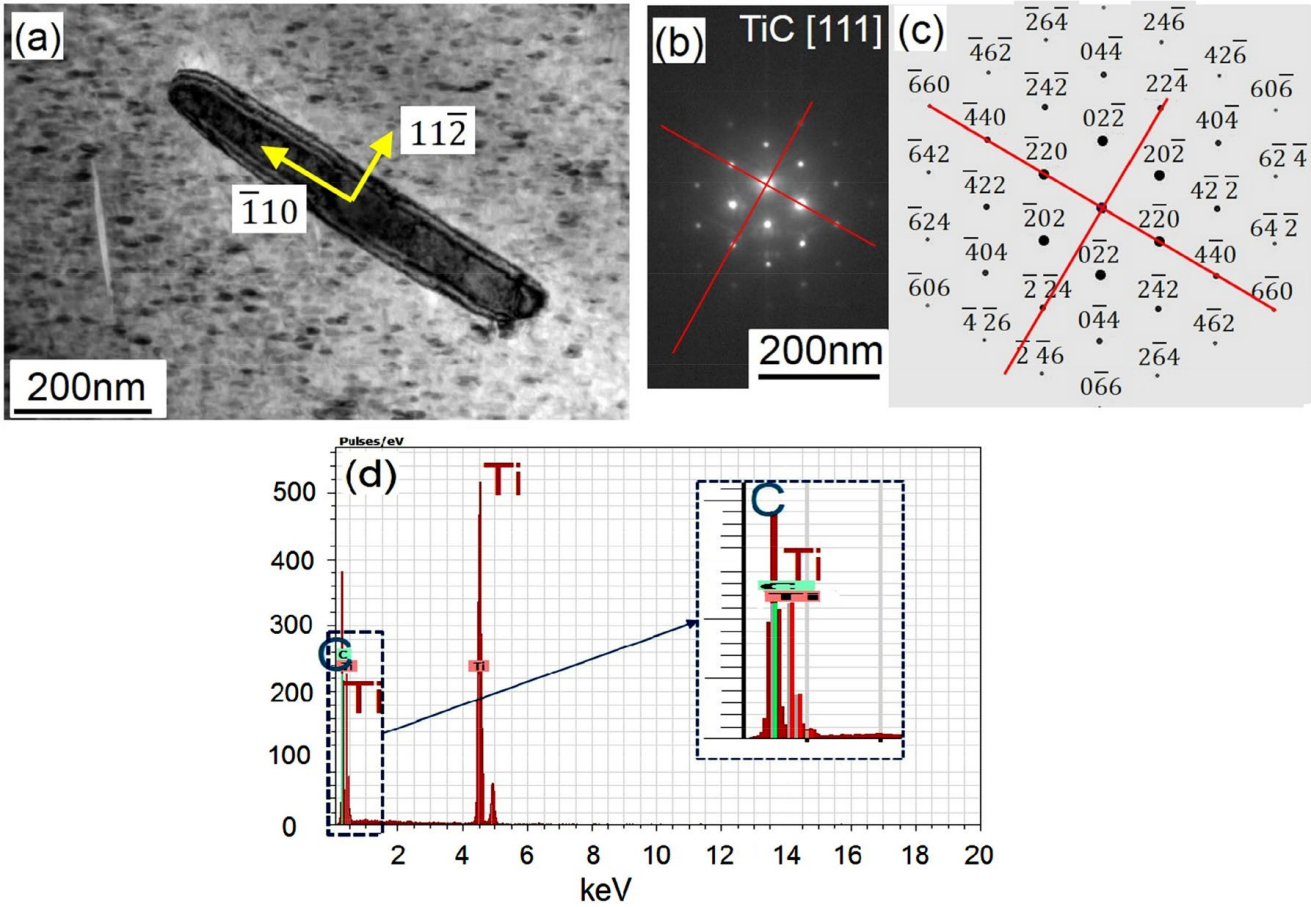
TEM observation of (**a**) TiC particle, growing in < 110 > direction determined from (**b**) diffraction pattern of TiC [111] and (**c**) the corresponding simulation of image (**b**), while the particle is composed of (**d**) Ti and C.

TiC ideally develops with an octahedron shape under equilibrium condition due to its NaCl-type face centred cubic structure (with a unit cell of a TiC_6_ octahedron)^35–37^. However, in a non-equilibrium state, thermal and mass transportation in the melt also play an important role, enabling different growth kinetics and mechanisms and hence a variety of growth morphologies^36^. As a result, according to the laser parameters used in SLM, TiC can form with different shapes, such as laminated, octahedron, semi-spherical^38^, or even whisker^39^. In the current case, whisker-shaped^40^ TiC nano constituents has been formed, as clearly shown in Fig. 11a. This peculiar whisker-shape originates from the rapid growth mechanisms activated in the <110> direction, as shown in Fig. 11b, c. Therefore, in this case, it seems that carbon has diffused in TiC to pass through {110} planes which provides the lowest packing density and hence the easiest route compared to {111} and {100} TiC planes^41^.

**Figure 12:**
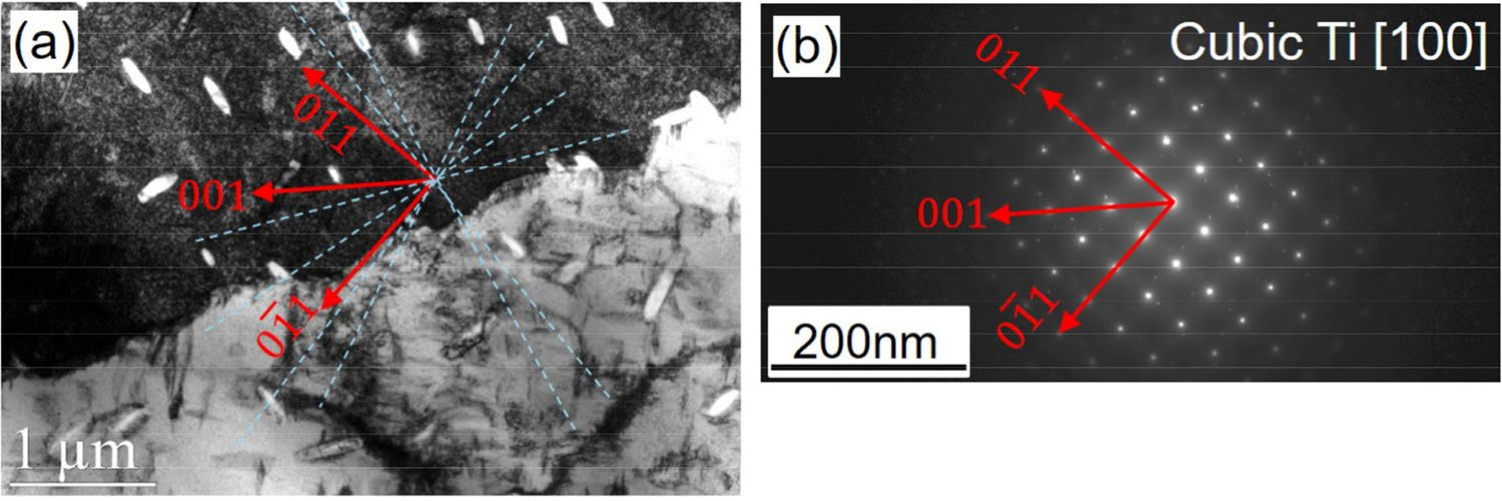
TEM observation of (**a**) TiC whiskers oriented in Ti grains and (**b**) dark field diffraction pattern of Ti [100] (top Ti grain), showing no certain orientation between the whiskers and the cubic Ti-matrix. The possible orientations of the whiskers across the grains are shown with dotted lined in (**a**).

It should be noted that no certain orientation relationship was found between the TiC whiskers and the main planes in the cubic Ti matrix (Fig. 12). No orientation relationship between the whiskers and the Ti matrix can be an indication for formation of these whiskers dominantly in the Ti melt. Accordingly, it seems that the TiC whiskers follow the Marangoni forces and the melt stream after the formation. The melt streams in irregular cycles towards the back of the laser melt pool where it solidifies. Within such streams there are strong velocities and temperature changes^33^. TiC whiskers, circulating with this stream, can logically leave the high velocity streams to lower velocity streams from several preferential locations in which the melt stream does a sharp turn. Some TiC whiskers can also be impeded at the solidified regions, orienting them into the liquid–solid borders according to their remaining momentum. After solidification, these lead to a Ti matrix homogeneously reinforced by numerous TiC whiskers with several preferential orientations across different grains and also congregated in grain boundaries (see Fig. 3). This is in agreement with the fact that particles can eject from the back of the laser melt track with some preferential orientations due to the melt pool dynamics^42^.

### Correlation of microstructure with mechanical properties

The microstructure of the in situ formed MMC parts from Ti/10.5 wt% Mo_2_C is a β-titanium matrix reinforced by homogenously distributed TiC whiskers with 50–200 nm width and a high aspect ratio, with lengths up to several micrometres (see Figs. 2, 3). β-Ti alloys are typically known to show lower hardness, strength and stiffness but at the same time a higher ductility than Ti–6Al–4V^17,18,43^. In this case, the ductile and low stiffness β-Ti matrix is reinforced with about 6 vol% of TiC whiskers. TiC is a high temperature ceramic with extremely high hardness (~ 3,200 HV) and elastic modulus (~ 448–451 GPa)^44^. TiC whisker, compared to its particulate shape, can provide more bonding interfaces. This can more effectively suppress the deformation of titanium, drastically increasing the hardness of reinforced β-Ti to about 513 ± 38 HV, the stiffness to about 126 ± 7 GPa (even higher than fully martensitic α-Ti), and the compressive strength to about 1642 ± 77 MPa. In return, this allows no plasticity (Figs. 5, 6) without deformation or displacement of ultra-strong TiC whiskers, owing to the excellent bonding between the TiC whiskers and the β-Ti matrix (see the well-bonded interfaces without any cracking, Fig. 11). As a result, the pressure under compressive deformation continues to grow until it reaches a value much greater than a common β-Ti matrix can sustain (~ 1642 MPa). At this stage, the β-Ti matrix catastrophically fails from the regions around the actual TiC whiskers where the deformation had been localised. As a result, compressive fracture appears as elongated and holed deformation cells which may have primary contained displaced TiC whiskers (Fig. 7b). This is of course very different with the smooth compressive fracture of pure Ti, as shown in Fig. 7a.

It is worth mentioning that the current Ti composite increases the strength of titanium without the use of any potentially harmful elements such as aluminium and vanadium. In contrary to Al and V, this work increases the hardness and strength of pure titanium through alloying with Mo (as a highly recommended element due to its high biocompatibility^23^) and generating nano TiC whisker reinforcements (TiC is an excellent constituent to improve the bioactivity of titanium^24,25^). Despite these very high hardness (over 500 HV) and strength (~ 1642 MPa true ultimate compression strength) of the developed Ti alloy composite (see Fig. 5), the absence of plasticity is unfortunately a real issue. The possible strategies such as use of less Mo_2_C content or application of heat treatment to improve this issue and to create a future bio-material are addressed in other works^45^. From the bio-aspect, another issue with this new composite material is the fact that stiffness is even more than Ti and Ti–6Al–4V (Fig. 5). Therefore, the material itself does not reduce the issue of stress shielding of the bone implants, however this can be easily improved by implementing porosity and lattice structure in design and manufacture of implants using AM^43,46^.

## Conclusions

This work developed a strong and hard Ti alloy composite without potentially cytotoxic elements such as Al and V using the SLM process from an in situ reactive Ti/10.5 wt% Mo_2_C powder mixture. It was found that:

- Despite the high cracking susceptibility, dense and crack free titanium alloy composites can be made via SLM from the Ti/10.5 wt% Mo_2_C powder mixture.
- After SLM of the Ti/Mo_2_C powder mixture, the in situ released Mo in conjunction with SLM rapid solidification stabilised the β-Ti phase (instead of martensitic α-phase). At the same time, this β-Ti matrix was homogenously reinforced with in situ formed and high aspect ratio TiC whiskers (50–200 nm wide and up to several micrometre long).
- During the SLM process, Mo_2_C progressively decomposed to Mo and carbon in the Ti melt. Mo, on one hand, diffused and dissolved in the Ti melt, while carbon nano particles migrated until they formed in situ TiC whiskers. This means that the in situ formation and growth mechanisms were controlled by decomposition, dissolution, migration and reformation of constituents.
- The whisker shape of the in situ formed TiC constituents was associated with the directional crystal growth along <110> direction. The distribution and orientation of TiC whiskers was argued to be dictated by the melt flow streams.
- TiC whiskers (as an extremely hard and strong material) established an excellent interfacial bonding with β-Ti matrix. As a result, the developed in situ TiC whisker reinforced β-Ti had a high hardness of 513 HV and the compressive strength of about 1642 MPa. The obtained Young’s modulus of 126 GPa was higher than that of fully martensitic α-Ti in as-SLM Ti or Ti–6Al–4V despite the low stiffness β-matrix.
- Despite the positive influence of TiC on the strength and the hardness, plastic deformation was hindered by the TiC whiskers in the β-Ti matrix, resulting in a catastrophic failure under very high stresses. Improving the extreme brittleness using appropriate heat treatments will be presented in other research works.

## Materials and methods

### Materials and SLM

**Figure 13:**
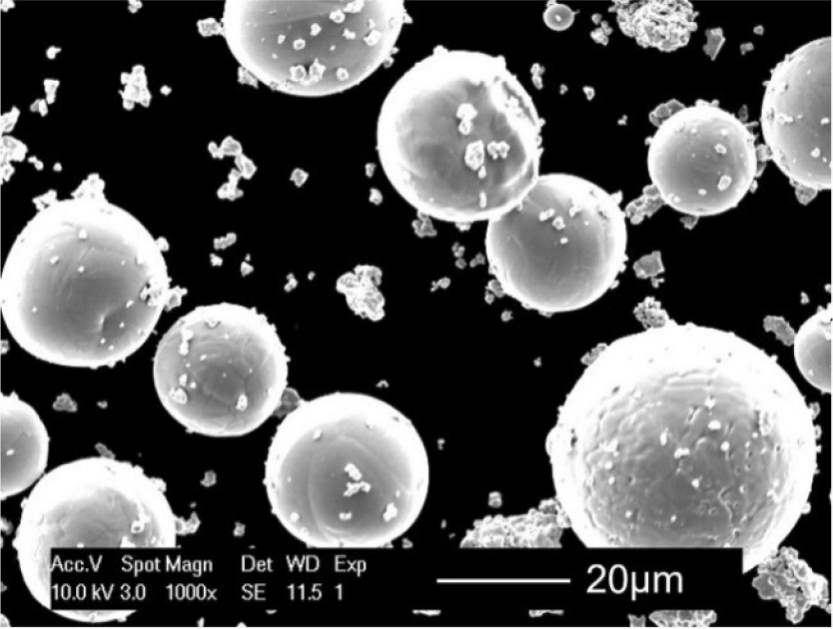
Powder mixture of Ti/10.5 wt% Mo_2_C. Cp-Ti is the larger spherical particles and Mo_2_C is the smaller and partially agglomerated particles.

Gas atomised Cp-Ti powder (grade 1) was obtained from LPW Ltd (UK) with a particle size ranging from 15 to 45 μm. The Ti powder was mixed with 10.5 wt% molybdenum carbide powder (Mo_2_C, supplied from Changsha Langfeng, China) with an average powder particle size ~ 3.5 µm, as seen in Fig. 13. Mo_2_C powder can be decomposed (i.e., broken down into Mo and C as the original constituents) in intermediate steps at higher temperatures, where there is a chance for the dissolution of Mo in Ti and the formation of more stable carbides such as TiC (which is also a fully biocompatible material^24,25^). This amount of Mo_2_C would theoretically generate around 9.88 wt% Mo and 0.62 wt% C after the decomposition. Since 10 wt% Mo was expected to be adequate to make a fully β-Ti matrix after rapid solidification^18^, this specific composition was chosen. This powder system was dry mechanically mixed in a Turbula mixer for at least 8 h. After mixing, although the Mo_2_C powder particles could fill the spaces between spherical Ti powders, the agglomeration of Mo_2_C particles (which is common for fine powders) remained to some extent after mixing (Fig. 13) and reduced the flowability, but it did not impede the SLM processing.

**Figure 14:**
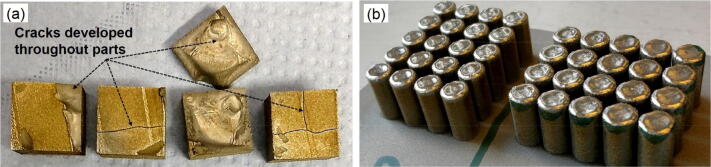
SLM part from powder mixture of Ti/10.5 wt% Mo_2_C; (**a**) Visual cracks growing in the parts, and (**b**) possibility of crack free production of components. *Note* the coloured surfaces at the bottom of the parts in figure a is only due to cutting by wire electro discharge machining.

A commercial 3DS ProX 320 SLM machine with 400 W fibre laser and a beam diameter of about 60 µm was used to manufacture samples. The SLM was carried out under a protective argon atmosphere keeping the oxygen below 100 ppm during the process. The laser power, scan speed, and hatch spacing were varied while the layer thickness was kept constant at 30 µm. Each layer was scanned once. A bi-directional scanning was carried out and a 90° rotation was applied between the layers. Optimisations were based on reaching the highest density as well as the absence of macrocracks. This was since the Ti/10.5 wt% Mo_2_C was extremely susceptible to cracking, as visually observed in Fig. 14a. Optimal parameters to produce dense and crack-free components were found (Fig. 14b) for both the Ti and Ti/Mo_2_C material systems, as summarised in Table 1.

**Table 1:** Optimum parameters resulting in crack free parts with over 99% density.

Material system	Laser power (P) (W)	Scanning speed (v) (mm/s)	Hatch spacing (h) (mm/s)
Ti/10.5 wt% Mo_2_C	300	1,000	70
Cp-Ti	235	1,400	70

### Characterisation

To investigate the degree of densification, the polished cross-sections were viewed using an Axiocam Leica optical microscope after polishing. All the cross-sections were cut and prepared along the SLM building direction (side view). To analyse the microstructure, a FEI-Nova Nanosem 450 equipped with EBSD detector was used to reveal the grain structures and the very fine secondary phases. To quantitatively analyse the features such as porosity or volume fraction of a phase, an average of at least 6 readings were recorded on up to 3 pictures using an image processing software (ImageJ). Moreover, transmission electron microscopy (TEM) was performed using a FEI Tecnai TEM at 200 kV, equipped with a Nanomegas ASTAR system and a Bruker XFlash 6T160 EDX unit. TEM samples were prepared using mechanical polishing and PIPS ion thinning. Phase identification was carried out by a Seifert 3,003 TT X-ray diffractometer (XRD) with coupled Theta/2Theta scan type and Cu-Kα1 radiation (wavelength: 0.15418 nm) operated at 40 kV and 40 mA.

Vickers microhardness tests were performed using a Future Tech FV-700 hardness tester with an indentation load of 1 kg. Compression tests were carried out to determine the strength of the material using a 250 kN INSTRON universal testing machine at room temperature and hardened tool steel as compressive plates. Tests were carried out parallel to the SLM building direction (vertical). The compression samples were cylindrical with 6 mm diameter and about 10 mm height. The top and bottom surfaces were cut by EDM beforehand and zinc stearate was used as lubricant. It should be noted that no extensometer could be used during compression and hence the deformation of the compression steel plates (present at the top and bottom of the samples) could also contribute to the measured strains. Therefore, the strains were not accurate (especially at elastic regions) and were only used for comparison purposes.

The impulse excitation technique (IET) was used to measure the Young's Modulus using a Resonance Frequency and Damping Analyser (RFDA, IMCE, Belgium) on machined bars with dimensions of about 2 mm × 6 mm × 60 mm, as described in ^47^.

## Data availability statement

Microstructural data generated or analysed during this study are included in this published article. Extra and raw data are available on reasonable request from the corresponding author.
